# Needlestick injuries: a prickly need for improving prevention

**DOI:** 10.1186/1753-6561-5-S6-P222

**Published:** 2011-06-29

**Authors:** I Agreiter, L Pagani, E Motter, E Pedrotti, P Mian

**Affiliations:** 1 Bolzano Central Hospital, Infectious Disease Unit, Bolzano, Italy

## Introduction / objectives

Health Care Workers (HCWs) are at high risk for needlestick injuries (NI). Many NI occur because of inappropriate management of devices or instruments. Improved education and appropriate sharps disposal after use would help reduce NI for HCWs involved in everyday practice.

## Methods

The Infectious Diseases Unit at Bolzano Hospital is the point of reference for all NI occurring in- and outside the hospital within the sanitary district (approx. 215,000 inhabitants). For each case noticed, a record form is drawn up by nurses, who take care of registration and demographics, occupation of the worker involved, timing of NI, and by physicians thereafter, who record cause of the accident and causal device, and manage potential exposure to bloodborne pathogens. All notified cases of NI occurring from 2003 to 2010 were recorded and analyzed.

## Results

Overall, 327 accidental NI were recorded. Twenty-six % were NI by insulin or intramuscular needles, 18% were represented by neglected needles, 14% by glycostix or glycemia lancets. 13% of the incidents occurred with surgical instruments, 10% during blood drawing, 6% by excessively full sharps-disposal containers. A fewer number of cases (<5% each) was represented by injuries during placement of intravenous cannulae, “cobra effect” of butterfly needles after blood drawing, recapping, or wrongly disposed needles. A worrying increase of NI has been recorded in the last years, mostly attributable to increased turnover of inadequately trained HCWs.

## Conclusion

Improper use of instruments or oversight of sharp-disposal containers by HCWs often causes incidents which would easily be prevented. Appropriate management of sharps is very important and needs continuous education and training not only at any HCWs level, but also for other employees in public utility jobs.

## Disclosure of interest

None declared.

